# Exposure to weak opioids and risk of gastrointestinal tract cancers: A series of nested case‐control studies

**DOI:** 10.1111/bcp.15759

**Published:** 2023-05-18

**Authors:** Martin G. Houston, Úna McMenamin, Brian Johnston, Ronald D. McDowell, Carmel M. Hughes, Peter Murchie, Chris R. Cardwell

**Affiliations:** ^1^ Centre for Public Health Queen's University Belfast Co. Antrim UK; ^2^ Department of Gastroenterology Royal Victoria Hospital Belfast Co. Antrim UK; ^3^ School of Pharmacy Queen's University Belfast Co. Antrim UK; ^4^ Institute of Applied Health Sciences Section Academic Primary Care Aberdeen UK

**Keywords:** codeine, colorectal cancer, gastric cancer, gastrointestinal motility, gastrointestinal neoplasms, oesophageal cancer, opioids

## Abstract

**Aims:**

There is evidence gastrointestinal (GI) motility may play a role in the development of GI cancers. Weak opioids (codeine and dihydrocodeine) decrease GI motility, but their effect on GI cancer risk has not been assessed. We aim to assess the association between weak opioids and cancers of the GI tract.

**Methods:**

A series of nested case‐control studies was conducted using Scottish general practice records from the Primary Care Clinical Informatics Unit Research database. Oesophageal (n = 2432), gastric (n = 1443) and colorectal cancer (n = 8750) cases, diagnosed between 1999 and 2011, were identified and matched with up to five controls. Weak opioid use was identified from prescribing records. Odds ratios (ORs) and 95% confidence intervals (CIs) were calculated using conditional logistic regression, adjusting for relevant comorbidities and medication use.

**Results:**

There was no association between weak opioids and colorectal cancer (adjusted OR = 0.96, CI 0.90, 1.02, *P* = 0.15). There was an increased risk of oesophageal (adjusted OR = 1.16, CI 1.04, 1.29, *P* = 0.01) and gastric cancer (adjusted OR = 1.26, CI 1.10, 1.45, *P* = 0.001). The associations for oesophageal cancer, but not gastric cancer, were attenuated when weak opioid users were compared with users of another analgesic (adjusted OR = 1.03 CI 0.86, 1.22, *P* = 0.76 and adjusted OR = 1.29 CI 1.02, 1.64, *P* = 0.04 respectively).

**Conclusions:**

In this large population‐based study, there was no consistent evidence of an association between weak opioids and oesophageal or colorectal cancer risk, but a small increased risk of gastric cancer. Further investigation is required to determine whether this association is causal or reflects residual confounding or confounding by indication.

**What is already known about this subject**

- A previous large cohort study of a Danish population has identified an increased risk of upper gastrointestinal (GI) cancer, but not colorectal cancer, in patients with constipation. Weak opioids are commonly prescribed drugs that decrease GI motility and cause constipation, but their impact on GI cancer has not been previously assessed.

**What this study adds**

- To the authors' knowledge, this is the first pharmacoepidemiological study that has examined weak opioid use and risk of GI malignancy. We observed an association between weak opioid use and gastric cancer risk, but no association with oesophageal or colorectal cancer risk.

## 1 INTRODUCTION

Gastrointestinal (GI) motility may play a role in the development of GI tract cancers. A recent, large Danish cohort study has demonstrated increased risk of various GI tract cancers in patients diagnosed with constipation. Although there was no long‐term risk of colorectal cancer, an increased risk of oesophageal, stomach, small intestine, liver and pancreatic cancer was observed after 15 years of follow‐up.^1^ Meta‐analysis of observational studies has provided conflicting evidence on the role of constipation and colorectal cancer risk.^2^ Regular exercise is associated with reduced GI cancer risk, potentially due to decreased GI transit time and subsequent reduced carcinogen exposure to GI mucosa. Several studies have demonstrated exercise also beneficially modifies the GI microbiome, although the underlying mechanisms remain unknown.^3^ Decreased GI motility due to opioid use has been associated with decreased GI mucosal integrity and subsequent dysbiosis,^4^ which is implicated in the development of GI cancers.^5^ Furthermore, there is experimental evidence that delayed gastric emptying increases risk of gastric cancer in murine models. Mice who underwent vagotomy (which delays gastric emptying) had an increased risk of gastric cancer following exposure to the carcinogen N‐methyl‐N′‐nitro‐N‐nitrosoguanidine. However, when combined with a drainage procedure such as pyloroplasty, thereby improving gastric emptying, risk of gastric cancer was decreased in vagotomised mice.^6^

Codeine and dihydrocodeine are widely prescribed opioid analgesics within the UK.^7^ Both drugs are classed as weak opioids in the British National Formulary^8^ and are used for mild to moderate pain on the World Health Organization's analgesic ladder.^9^ Opioids bind to mu receptors in the GI tract and decrease motility by inhibiting cholinergic neurotransmission,^10^ and constipation is a well‐documented side‐effect in primary care.^11^ Codeine has been shown in human studies to decrease oesophageal peristalsis,^12^ delay gastric emptying^13^ and increase colonic transit time.^14^

To date, there has not been a study that has investigated the effect of weak opioids on risk of developing GI malignancy. Given their common usage and substantial effect on GI motility, we investigated the association between weak opioids and the risk of oesophageal, gastric and colorectal cancer in a series of nested case‐control studies within a large population‐based general practice database.

## 2 PATIENTS AND METHODS

### 2.1 Data source

The study was conducted using data from the Primary Care Clinical Information Unit Research (PCCIUR) database.^15^ The PCCIUR captures information from General Practice records including demographics, diagnoses, prescriptions and lifestyle characteristics (including smoking and alcohol intake), and has been used extensively for research.^16^, ^17^, ^18^, ^19^ The PCCIUR contained over two million patients registered at 393 general practices in Scotland between 1993 and 2011. Data access was approved by the Research Applications and Data Management Team of the University of Aberdeen.

### 2.2 Study design

A series of nested case‐control studies was conducted within the PCCIUR database. New cases of oesophageal, gastric and colorectal cancer, diagnosed between 1999 and 2011, were identified using General Practice Read codes. Cases were excluded if they had a diagnosis of another cancer, apart from nonmelanoma skin cancer, on or before the date of their GI cancer diagnosis. Each case was matched with up to five controls based on gender, GP practice, year of birth plus‐or‐minus 5 years and year of registration (in categories). The date of cancer diagnosis was set as the index date for each case as well as their matched controls. Each control had to be alive and free from cancer, excluding nonmelanoma skin cancer, and registered with their GP on the index date. Cases and controls were excluded if they did not have at least 3 years of continuous primary care records with the same general practice prior to the index date.

**FIGURE 1:**
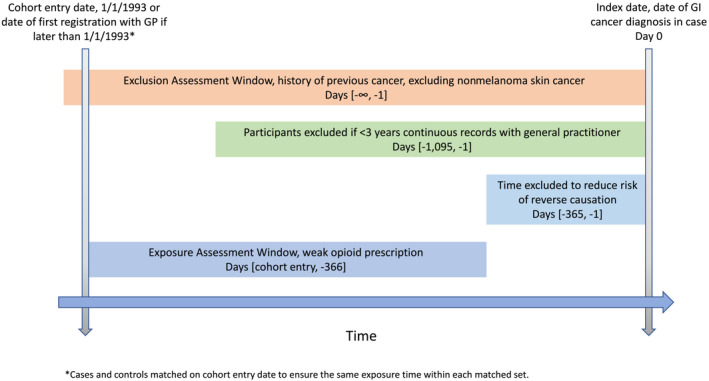
Exposure period of cases and controls assessed in main analysis.

Within each matched set, the exposure period began on either 1 January 1993 (as the electronic prescription records are less likely to be complete before this time) or the most recent GP registration date within the matched set if this occurred after 1 January 1993. This method ensured that the exposure period was the same for cases and controls within each matched set. The exposure period finished 1 year before the index date to reduce the risk of reverse causation as medications taken during this period are unlikely to have contributed to carcinogenesis (Figure 1).

### 2.3 Exposure

We ascertained medication use from each individual prescription within the exposure period as classified in the British National Formulary.^8^ We identified codeine prescriptions (including codeine alone and codeine with other medications; 96% of codeine prescriptions were a codeine and paracetamol compound medication) and dihydrocodeine prescriptions (including dihydrocodeine alone and dihydrocodeine combined with other medications; 62% of dihydrocodeine prescriptions were a dihydrocodeine and paracetamol compound medication). We also identified prescriptions for ibuprofen and paracetamol, commonly prescribed nonopioid analgesics, to act as active comparators.

### 2.4 Covariates

Relevant comorbidities were identified from published Read codes^20^ to include in our analysis. We included the following comorbidities from the Charlson Comorbidity Index in all analyses: myocardial infarction, ischaemic heart disease, heart failure, peripheral vascular disease, dementia, cerebrovascular disease, chronic pulmonary disease, peptic ulcer, rheumatological disease, HIV status and renal disease. We included these covariates in our adjusted model as markers for general health to reduce the risk of confounding. Additionally, inflammatory bowel disease was included in the model for colorectal cancer as it is a known risk factor.^21^ Medications which may have a preventative effect on GI tract cancer were incorporated into the model in all analyses, namely aspirin, statins and nonsteroidal anti‐inflammatory drugs (NSAIDs).^22^, ^23^, ^24^, ^25^ The Scottish Index of Multiple Deprivation based on the postcode of the GP practice was determined as a measure of deprivation.^26^

### 2.5 Statistical analyses

Characteristics of cases and controls were compared using frequencies and percentages for qualitative variables and descriptive statistics for continuous variables. We applied conditional logistic regression to calculate odds ratios (ORs) and 95% confidence intervals (CIs) for associations between weak opioids (either of codeine and/or dihydrocodeine) and oesophageal, gastric and colorectal cancer. The matched design accounted for GP practice, sex, year of registration and age in categories, and, in addition, age in years was entered into both the unadjusted and adjusted models. We investigated use of weak opioids (including codeine or dihydrocodeine) and codeine and dihydrocodeine separately. We also investigated the number of prescriptions and timing of prescriptions (ie, in the year immediately before cancer diagnosis, in the 1‐to‐2 year period before cancer diagnosis, 2‐to‐3 year period and greater than 3 years prior). This allowed us to assess the effect of reverse causality in the year immediately before diagnosis (as this time period is excluded from the main analysis) as well as look for any persistent temporal association between weak opioid use and cancer risk.

We performed a number of further analyses. First, two active comparator analyses were conducted (to attempt to reduce confounding by indication),^27^ one comparing weak opioid users to ibuprofen users (no adjustment was made for NSAIDs in this analysis) and another comparing weak opioid users to paracetamol users who had not used weak opioids. We performed an analysis additionally adjusting for smoking and alcohol using a complete case approach and a multiple imputation approach. In the multiple imputation approach, smoking was imputed based on an ordinal logistic regression model including case status and all covariates from the model, including weak opioids. Twenty‐five imputations^28^ were conducted and results were combined using Rubin's rules.^29^ This approach was used for smoking, alcohol, and both smoking and alcohol. We repeated the main analysis extending the lag period to 2 years to further reduce the risk of reverse causation. Finally, we conducted separate analyses of paracetamol prescriptions (ie, excluding prescriptions containing weak opioids) and ibuprofen prescriptions to investigate pain medications, in general, on GI cancer risk. All statistical analyses were conducted using STATA 16 (StataCorp, College Station, TX, USA).

## 3 RESULTS

### 3.1 Characteristics of cases and controls

Characteristics of cases, controls and selected comorbidities are summarized in Table 1. A total of 2432 oesophageal, 1443 gastric and 8750 colorectal cancer cases were matched with 10 590, 6233 and 38 264 controls respectively. In all three cancer sites, most cases were diagnosed between the ages of 70 and 79 years old and more cases were male. Smoking and alcohol consumption (where data was available) were similar between cases and controls.

**TABLE 1:** Characteristics of cases and controls.

Cancer site	Oesophageal		Gastric		Colorectal	
	Cases	Controls	Cases	Controls	Cases	Controls
	n = 2432	n = 10 590	n = 1443	n = 6233	n = 8750	n = 38 264
Age, mean (SD)	69.1 (11.4)	66.5 (12.1)	71.2 (11.3)	68.9 (12.0)	69.6 (11.6)	67.2 (12.3)
<50	118 (4.9%)	869 (8.2%)	63 (4.4%)	401 (6.4%)	471 (5.4%)	3132 (8.2%)
50‐59	394 (16.2%)	2322 (21.9%)	146 (10.1%)	915 (14.7%)	1192 (13.6%)	7005 (18.3%)
60‐69	664 (27.3%)	2923 (27.6%)	370 (25.6%)	1712 (27.5%)	2378 (27.2%)	10 887 (28.5%)
70‐79	802 (33.0%)	2825 (26.7%)	507 (35.1%)	1989 (31.9%)	2916 (33.3%)	10 850 (28.4%)
>80	454 (18.7%)	1651 (15.6%)	357 (24.7%)	1216 (19.5%)	1793 (20.5%)	6390 (16.7%)
Gender						
Male	1645 (67.6%)	7185 (67.8%)	827 (57.3%)	3539 (56.8%)	4795 (54.8%)	20 731 (54.2%)
Deprivation (in quintiles)						
1st (most deprived)	682 (28.0%)	2950 (27.9%)	422 (29.2%)	1820 (29.2%)	2226 (25.4%)	9637 (25.2%)
2nd	601 (24.7%)	2619 (24.7%)	383 (26.5%)	1648 (26.4%)	2239 (25.6%)	9761 (25.5%)
3rd	405 (16.7%)	1791 (16.9%)	241 (16.7%)	1055 (16.9%)	1432 (16.4%)	6307 (16.5%)
4th	483 (19.9%)	2112 (19.9%)	263 (18.2%)	1146 (18.4%)	1840 (21.0%)	8108 (21.2%)
5th (least deprived)	254 (10.4%)	1087 (10.3%)	133 (9.2%)	559 (9.0%)	1003 (11.5%)	4410 (11.5%)
Missing	7 (0.3%)	31 (0.3%)	1 (0.1%)	5 (0.1%)	10 (0.1%)	41 (0.1%)
Smoking						
Never	633 (26.0%)	3623 (34.2%)	476 (33.0%)	2217 (35.6%)	3120 (35.7%)	13 371 (34.9%)
Former	539 (22.2%)	2079 (19.6%)	308 (21.3%)	1246 (20.0%)	1959 (22.4%)	7297 (19.1%)
Current	726 (29.9%)	2336 (22.1%)	349 (24.2%)	1398 (22.4%)	1657 (18.9%)	8143 (21.3%)
Missing	534 (22.0%)	2552 (24.1%)	310 (21.5%)	1372 (22.0%)	2014 (23.0%)	9453 (24.7%)
Alcohol						
None	365 (15.0%)	1413 (13.3%)	280 (19.4%)	999 (16.0%)	1330 (15.2%)	5738 (15.0%)
Low	1126 (46.3%)	5090 (48.1%)	655 (45.4%)	2966 (47.6%)	4265 (48.7%)	17 974 (47.0%)
High	152 (6.3%)	452 (4.3%)	44 (3.0%)	214 (3.4%)	328 (3.7%)	1292 (3.4%)
Missing	789 (32.4%)	3635 (34.3%)	464 (32.2%)	2054 (33.0%)	2827 (32.3%)	13 260 (34.7%)
Selected comorbidities						
Reflux oesophagitis	242 (10.0%)	530 (5.0%)	90 (6.2%)	356 (5.7%)	482 (5.5%)	1868 (4.9%)
Barrett's oesophagus	96 (3.9%)	60 (0.6%)	6 (0.4%)	48 (0.8%)	52 (0.6%)	214 (0.6%)
Peptic ulcer disease	327 (13.4%)	917 (8.7%)	252 (17.5%)	630 (10.1%)	839 (9.6%)	3205 (8.4%)
Diabetes mellitus	237 (9.7%)	900 (8.5%)	165 (11.4%)	537 (8.6%)	975 (11.1%)	3122 (8.2%)
Myocardial infarction	193 (7.9%)	764 (7.2%)	129 (8.9%)	493 (7.9%)	603 (6.9%)	2509 (6.6%)
IHD	455 (18.7%)	1695 (16.0%)	329 (22.8%)	1154 (18.5%)	1514 (17.3%)	6048 (15.8%)
Heart failure	109 (4.5%)	373 (3.5%)	64 (4.4%)	264 (4.2%)	350 (4.0%)	1289 (3.4%)
PAD	159 (6.5%)	468 (4.4%)	90 (6.2%)	311 (5.0%)	425 (4.9%)	1665 (4.4%)
IBD	141 (5.8%)	536 (5.1%)	85 (5.9%)	326 (5.2%)	502 (5.7%)	2027 (5.3%)
Selected medications						
Aspirin	739 (30.4%)	2904 (27.4%)	526 (36.5%)	1919 (30.8%)	2613 (29.9%)	10 447 (27.3%)
Statins^a^	573 (23.6%)	2182 (20.6%)	345 (23.9%)	1348 (21.6%)	1956 (22.4%)	7529 (19.7%)
NSAIDs^b^	1001 (41.2%)	4416 (41.7%)	600 (41.6%)	2658 (42.6%)	3525 (40.3%)	15 753 (41.2%)

Abbreviations: IBD, inflammatory bowel disease; IHD, ischaemic heart disease; NSAID, nonsteroidal anti‐inflammatory drug; PAD, peripheral arterial disease.

^a^ Atorvastatin, fluvastatin, pravastatin, rosuvastatin, simvastatin.

^b^ Aceclofenac, acemetacin, celecoxib, dexibuprofen, dexketoprofen, diclofenac, etodolac, etoricoxib, fenoprofen, ibuprofen, indomethacin, ketoprofen, mefenamic acid, meloxicam, nabumetone, naproxen, piroxicam, sulindac, tenoxicam, tiaprofenic acid, rofecoxib, valdecoxib, lumiracoxib.

### 3.2 Main analysis

#### 3.2.1 Weak opioids and oesophageal cancer risk

We observed a small positive association between weak opioids and risk of oesophageal cancer (see Table 2, adjusted OR = 1.16, CI 1.04, 1.29, *P* = 0.01). This did not follow an obvious dose response as the association was apparent in both those with least use, six prescriptions or fewer (adjusted OR = 1.18, CI 1.05, 1.34, *P* = 0.01), and those with highest use, more than 24 prescriptions (adjusted OR = 1.26, CI 1.02, 1.56, *P* = 0.04). Associations were similar for codeine and dihydrocodeine use (adjusted OR = 1.12, CI 1.00, 1.25, *P* = 0.05 and adjusted OR = 1.06, CI 0.92, 1.23, *P* = 0.43, respectively). The active comparator analysis showed there was no difference in oesophageal cancer risk in weak opioid users compared with ibuprofen users or paracetamol users. Furthermore, the association between weak opioids and oesophageal cancer was only apparent in the first 3 years before diagnosis. Associations were largely similar in sensitivity analyses (see Table 4).

**TABLE 2:** Exposure to weak opioids (codeine and dihydrocodeine) and risk of oesophageal and gastric cancer.

Medication	Cases	Controls	Age adjusted OR, 95% CI	Adjusted^a^ OR, 95% CI	Adjusted^a^ *P* value
Oesophageal cancer					
Weak opioids					
Nonuser	1563 (64.3%)	7217 (68.1%)	1.00 (ref. category)	1.00 (ref. category)	
User	869 (35.7%)	3373 (31.9%)	1.18 (1.06, 1.31)	1.16 (1.04, 1.29)	0.01
1‐6 prescriptions	548 (22.5%)	2123 (20.0%)	1.19 (1.06, 1.35)	1.18 (1.05, 1.34)	0.01
7‐24 prescriptions	170 (7.0%)	729 (6.9%)	1.04 (0.87, 1.25)	1.02 (0.84, 1.23)	0.87
>24 prescriptions	151 (6.2%)	521 (4.9%)	1.34 (1.09, 1.65)	1.26 (1.02, 1.56)	0.04
Weak opioid type (user *vs* nonuser)					
Codeine	700 (28.8%)	2723 (25.7%)	1.14 (1.02, 1.27)	1.12 (1.00, 1.25)	0.05
Dihydrocodeine	334 (13.7%)	1336 (12.6%)	1.11 (0.97, 1.28)	1.06 (0.92, 1.23)	0.43
Active comparator					
Ibuprofen users^b^	215 (8.8%)	923 (8.7%)	1.00 (ref. category)	1.00 (ref. category)	
Weak opioid users	869 (35.7%)	3373 (31.9%)	1.08 (0.91, 1.28)	1.03 (0.86, 1.22)	0.76
Paracetamol users^b^	152 (6.3%)	615 (5.8%)	1.00 (ref. category)	1.00 (ref. category)	
Weak opioid users	869 (35.7%)	3373 (31.9%)	1.22 (1.00, 1.50)	1.21 (0.99, 1.49)	0.07
Weak opioid use (by timing)					
0‐1 years prior^c^	629 (25.9%)	1868 (17.6%)	1.66 (1.49, 1.86)	1.63 (1.45, 1.83)	<0.001
1‐2 years prior	475 (19.5%)	1759 (16.6%)	1.19 (1.06, 1.35)	1.16 (1.02, 1.32)	0.02
2‐3 years prior	435 (17.9%)	1614 (15.2%)	1.20 (1.05, 1.36)	1.15 (1.01, 1.31)	0.03
>3 years prior^d^	600 (26.3%)	2390 (24.0%)	1.11 (0.99, 1.25)	1.08 (0.95, 1.22)	0.23
Gastric cancer					
Weak opioids					
Nonuser	866 (60.0%)	4087 (65.6%)	1.00 (ref. category)	1.00 (ref. category)	
User	577 (40.0%)	2146 (34.4%)	1.33 (1.17, 1.52)	1.26 (1.10, 1.45)	0.001
1‐6 prescriptions	325 (22.5%)	1291 (20.7%)	1.27 (1.09, 1.48)	1.22 (1.04, 1.43)	0.02
7‐24 prescriptions	127 (8.8%)	463 (7.4%)	1.30 (1.04, 1.62)	1.20 (0.95, 1.52)	0.12
>24 prescriptions	125 (8.7%)	392 (6.3%)	1.59 (1.26, 2.01)	1.50 (1.18, 1.90)	0.001
Weak opioid type (user *vs* nonuser)					
Codeine	476 (33.0%)	1734 (27.8%)	1.36 (1.19, 1.57)	1.29 (1.12, 1.50)	0.001
Dihydrocodeine	226 (15.7%)	849 (13.6%)	1.18 (0.99, 1.40)	1.10 (0.92, 1.32)	0.28
Active comparator					
Ibuprofen users^b^	109 (7.6%)	567 (9.1%)	1.00 (ref. category)	1.00 (ref. category)	
Weak opioid users	577 (40.0%)	2146 (34.4%)	1.39 (1.10, 1.76)	1.29 (1.02, 1.64)	0.04
Paracetamol users^b^	119 (8.2%)	429 (6.9%)	1.00 (ref. category)	1.00 (ref. category)	
Weak opioid users	577 (40.0%)	2146 (34.4%)	1.13 (0.89, 1.43)	1.09 (0.86, 1.39)	0.45
Weak opioid use (by timing)					
0‐1 years prior^c^	437 (30.3%)	1247 (20.0%)	1.83 (1.59, 2.10)	1.76 (1.52, 2.03)	<0.001
1‐2 years prior	341 (23.6%)	1177 (18.9%)	1.34 (1.16, 1.55)	1.27 (1.09, 1.48)	0.002
2‐3 years prior	306 (21.2%)	1062 (17.0%)	1.30 (1.12, 1.52)	1.22 (1.04, 1.43)	0.02
>3 years prior^d^	420 (30.8%)	1557 (26.4%)	1.28 (1.11, 1.48)	1.21 (1.04, 1.41)	0.01

Abbreviations: CI, 95% confidence interval; OR, odds ratio, 95%.

^a^ Individually adjusted for comorbidities in the Charlson Comorbidity Index; peptic ulcer disease, diabetes, myocardial infarction, heart failure, peripheral arterial disease, cerebrovascular disease, dementia, chronic obstructive pulmonary disease, connective tissue disease, liver disease, renal disease, HIV/AIDS, and aspirin, statin, and nonsteroidal anti‐inflammatory drug use (latter excluded in weak opioids/ibuprofen comparison).

^b^ Excludes weak opioid users.

^c^ This time period is excluded from the main analysis.

^d^ Cases and controls excluded if less than 4 years of continuous records prior to index date: oesophageal cancer cases = 2285, controls = 9961; gastric cancer cases = 1363, controls = 5891.

#### 3.2.2 Weak opioids and gastric cancer risk

We observed a significant positive association between weak opioids and gastric cancer (see Table 2, adjusted OR = 1.26, CI 1.10, 1.45, *P* = 0.001). This appeared to follow an exposure response with individuals using more than 24 prescriptions having higher risk (adjusted OR = 1.50, CI 1.18, 1.90, *P* = 0.001). The associations were only apparent for codeine and not dihydrocodeine (adjusted OR 1.29, CI 1.12, 1.50, *P* = 0.001 and adjusted OR = 1.10, CI 0.92, 1.32, *P* = 0.28, respectively). In the active comparator analysis, weak opioid users had a higher risk of gastric cancer compared with ibuprofen users (adjusted OR = 1.29, CI 1.02, 1.64, *P* = 0.04) but not paracetamol users. The association between weak opioids and gastric cancer was more marked in the year prior to cancer diagnosis but was still detectable more than 3 years before diagnosis (adjusted OR = 1.21, CI 1.04, 1.41, *P* = 0.01), when the lag period was extended to 2 years, and when adjusted for smoking and alcohol use (see Table 4). A separate analysis of paracetamol excluding weak opioid use (see Supporting Information Table S1) showed a similar association with gastric cancer risk, with individuals receiving more than 24 prescriptions having a more marked increase in risk (adjusted OR = 1.87, CI 1.32, 2.65, *P* < 0.001).

#### 3.2.3 Weak opioids and colorectal cancer risk

Table 3 shows there was no evidence of an association between weak opioids and colorectal cancer (adjusted OR = 0.96, CI 0.90, 1.02, *P* = 0.15). The findings were similar by frequency of use, by weak opioid type and when active comparators were used. Findings were similar in sensitivity analyses (Table 4).

**TABLE 3:** Exposure to weak opioids (codeine and dihydrocodeine) and risk of colorectal cancer.

Medication	Cases	Controls	Age adjusted OR, 95% CI	Adjusted^a^ OR, 95% CI	Adjusted^a^ *P* value
Colorectal cancer					
Weak opioids					
Nonuser	5977 (68.3%)	26 147 (68.3%)	1.00 (ref. category)	1.00 (ref. category)	
User	2773 (31.7%)	12 117 (31.7%)	0.97 (0.92, 1.02)	0.96 (0.90, 1.02)	0.15
1‐6 prescriptions	1754 (20.0%)	7458 (19.5%)	1.02 (0.95, 1.08)	1.01 (0.94, 1.07)	0.87
7‐24 prescriptions	560 (6.4%)	2648 (6.9%)	0.86 (0.78, 0.95)	0.85 (0.76, 0.94)	0.002
>24 prescriptions	459 (5.2%)	2011 (5.3%)	0.93 (0.83, 1.04)	0.90 (0.80, 1.01)	0.08
Weak opioid type (user *vs*. non‐user)					
Codeine	2271 (26.0%)	9874 (25.8%)	0.97 (0.91, 1.03)	0.96 (0.90, 1.02)	0.20
Dihydrocodeine	1052 (12.0%)	4749 (12.4%)	0.95 (0.88, 1.03)	0.94 (0.87, 1.02)	0.16
Active comparator					
Ibuprofen users^b^	788 (9.0%)	3283 (8.6%)	1.00 (ref. category)	1.00 (ref. category)	
Weak opioid users	2773 (31.7%)	12 117 (31.7%)	0.93 (0.84, 1.01)	0.91 (0.83, 1.00)	0.05
Paracetamol users^b^	616 (7.0%)	2282 (6.0%)	1.00 (ref. category)	1.00 (ref. category)	
Weak opioid users	2773 (31.7%)	12 117 (31.7&)	0.94 (0.85, 1.04)	0.92 (0.83, 1.02)	0.12
Weak opioid use (by timing)					
0‐1 years prior^c^	2084 (23.8%)	7051 (18.4%)	1.39 (1.31, 1.48)	1.40 (1.31, 1.49)	<0.001
1‐2 years prior	1516 (17.3%)	6396 (16.7%)	1.01 (0.95, 1.08)	1.00 (0.93, 1.07)	0.97
2‐3 years prior	1356 (15.5%)	5894 (15.4%)	0.97 (0.91, 1.04)	0.96 (0.90, 1.03)	0.30
>3 years prior^d^	1941 (23.8%)	8700 (24.4%)	0.92 (0.87, 0.98)	0.91 (0.85, 0.97)	0.01

Abbreviations: 95% CI, 95% confidence interval; OR, odds ratio.

^a^ Individually adjusted for comorbidities in the Charlson Comorbidity Index; peptic ulcer disease, diabetes, myocardial infarction, heart failure, peripheral arterial disease, cerebrovascular disease, dementia, chronic obstructive pulmonary disease, connective tissue disease, liver disease, renal disease, HIV/AIDS, inflammatory bowel disease, and aspirin, statin, and nonsteroidal anti‐inflammatory drug use.

^b^ Excludes weak opioid users.

^c^ This time period is excluded from the main analysis.

^d^ Cases and controls excluded if less than 4 years of continuous records prior to index date: colorectal cancer cases = 8162, controls = 35 726.

**TABLE 4:** Sensitivity analyses, weak opioids and gastrointestinal cancer risk.

Medication exposure	Cases	Controls	Adjusted^a^ OR, 95% confidence limits	Adjusted^a^ *P* value
Oesophageal cancer				
Weak opioids (primary analysis)	2432	10 590	1.16 (1.04, 1.29)	0.01
Weak opioids (2‐year lag period)	2285	9961	1.12 (1.00, 1.26)	0.04
Additionally adjusted for smoking using complete case	1898	8038	1.15 (1.01, 1.30)	0.03
Additionally adjusted for smoking using multiple imputation	2432	10 590	1.14 (1.02, 1.27)	0.03
Additionally adjusted for alcohol using multiple imputation	2432	10 590	1.15 (1.03, 1.29)	0.01
Adjusting for smoking and alcohol using multiple imputation	2432	10 590	1.13 (1.01, 1.26)	0.03
Gastric cancer				
Weak opioids (primary analysis)	1443	6233	1.26 (1.10, 1.45)	0.001
Weak opioids (2‐year lag period)	1363	5891	1.27 (1.10, 1.47)	0.001
Additionally adjusted for smoking using complete case	1133	4861	1.25 (1.07, 1.47)	0.01
Additionally adjusted for smoking using multiple imputation	1443	6233	1.25 (1.09, 1.44)	0.002
Additionally adjusted for alcohol using multiple imputation	1443	6233	1.25 (1.09, 1.44)	0.002
Adjusting for smoking and alcohol using multiple imputation	1443	6233	1.24 (1.08, 1.43)	0.003
Colorectal cancer				
Weak opioids (primary analysis)	8750	38 264	0.96 (0.90, 1.02)	0.15
Weak opioids (2‐year lag period)	8162	35 726	0.96 (0.90, 1.01)	0.14
Additionally adjusted for smoking using complete case	6736	28 811	0.94 (0.88, 1.00)	0.06
Additionally adjusted for smoking using multiple imputation	8750	38 264	0.96 (0.90, 1.02)	0.18
Additionally adjusted for alcohol using multiple imputation	8750	38 264	0.96 (0.90, 1.02)	0.17
Adjusting for smoking and alcohol using multiple imputation	8750	38 264	0.96 (0.91, 1.03)	0.22

Abbreviations: 95% CI, 95% confidence interval; OR, odds ratio.

^a^ Individually adjusted for comorbidities in the Charlson Comorbidity Index; peptic ulcer disease, diabetes, myocardial infarction, heart failure, peripheral arterial disease, cerebrovascular disease, dementia, chronic obstructive pulmonary disease, connective tissue disease, liver disease, renal disease, HIV/AIDS, and aspirin, statin, and nonsteroidal anti‐inflammatory drug use. Inflammatory bowel disease was also included in the model for colorectal cancer.

## 4 DISCUSSION

In our study, we observed no consistent evidence of an association between weak opioids and oesophageal and colorectal cancer, but some evidence of association between weak opioids and gastric cancer. The gastric cancer risk appeared to follow an exposure response and remained when compared with ibuprofen, but was attenuated when compared to paracetamol and was similar to the association between paracetamol use and gastric cancer risk.

The cause of the association between weak opioids and gastric cancer is unknown. It could reflect our hypothesis that a decrease in GI motility increases risk of GI tract cancers. We chose to study weak opioids due to their well‐documented side‐effects of constipation^30^ and their common usage in the UK.^7^ There is also evidence of a direct effect of codeine on oesophageal peristalsis,^12^ gastric emptying^13^ and colonic transit^14^ in human studies. In support of this we observed an exposure response, and we observed an increased risk of gastric cancer in weak opioid users compared with ibuprofen users (who may share indications). Opioids have also been shown in experimental models to affect the integrity of GI epithelial cells^31^ and increase pro‐inflammatory cytokines through induction of the immune system.^32^ Alternatively, the gastric cancer association could reflect confounding by indication and there was some evidence of this as the association between paracetamol, used for pain, and gastric cancer was similar to the association for weak opioids. Future studies of weak opioids and gastric cancer are warranted and should attempt to account for chronic pain.

The lack of association between weak opioids and oesophageal and colorectal cancer is reassuring to clinicians and patients. Weak opioids provide pain relief for mild to moderate pain in both acute and chronic settings and are included in the World Health Organization's model list of essential medicines.^33^

Previous studies have provided some evidence for decreased GI motility and GI cancer risk. A 2019 study of a large Danish cohort by Sundbøll et al found patients with constipation had increased risk of oesophageal, stomach, small intestinal, liver and pancreatic cancer at 15 years of follow‐up; the authors posited that delayed motility may lead to dysbiosis of the GI flora, with toxic bacterial metabolites able to disseminate throughout the body.^1^ Increased transit time may also be harmful by increasing exposure time of ingested or endogenously produced carcinogens to the GI mucosa; this has been suggested as a possible mechanism in the development of colorectal cancer,^34^ but the evidence for constipation as a risk factor for colorectal cancer is conflicting.^2^ Decreased GI motility has also been implicated in breast cancer, with the underlying mechanism thought to be decreased rate of oestrogen excretion from the increased GI transit time.^35^ Conversely, exercise may decrease cancer risk by decreasing transit time and having a positive effect on gut microbiota composition.^3^, ^36^

Our study has strengths and weaknesses. To our knowledge, this is the first study to focus on weak opioids and GI cancer. The PCCIUR is population‐based and captured prescription records for up to 18 years, eliminating the potential for recall bias. PCCIUR primary care records have been shown to be largely accurate at identifying cancer patients.^37^ We adjusted for a wide range of potential confounders, including smoking and alcohol, which may be particularly important for GI cancer risk^38^, ^39^ but we did not have access to others such as body mass index and *Helicobacter pylori* status and hence there remains the possibility of residual confounding. We did not have cancer registry records to investigate GI cancer by histological subtype.^40^, ^41^ We also did not have access to over‐the‐counter medication usage but codeine and dihydrocodeine are only available over the counter in the UK at low doses and with restricted pack sizes.^42^, ^43^ We also note that our comparator medications are available over the counter. However, methodological studies have shown that prescription data can give valid estimates of association even when medications are available over the counter.^44^ It is possible that the observed association for weak opioids and gastric cancer could reflect type I error. Finally, these results are not independent of an earlier screening study^16^ using the PCCIUR database which observed in one analysis an association between codeine and gastric cancer, but that previous study did not investigate weak opioids, did not investigate the timing of medication use and did use active comparators to compare weak opioids with other pain medications.

## 5 CONCLUSION

We observed no consistent evidence of an association between weak opioids and an increased risk of oesophageal and colorectal cancer, but some evidence of a small association between weak opioids and gastric cancer. Opioids remain useful analgesics; further studies are required to replicate these findings, both for opioids and other medications which affect GI motility, to help inform clinicians' safe prescribing practice.

## AUTHOR CONTRIBUTIONS

Funding acquisition: Chris R. Cardwell, Carmel M. Hughes and Peter Murchie. Data acquisition: Chris R. Cardwell, Ronald D. McDowell, Carmel M. Hughes and Peter Murchie. Study design: Martin G. Houston, Chris R. Cardwell, Ronald D. McDowell, Carmel M. Hughes and Peter Murchie. Data analysis and interpretation: Martin G. Houston, Chris R. Cardwell, Úna McMenamin, Brian Johnston, Ronald D. McDowell, Carmel M. Hughes and Peter Murchie. Writing original draft: Martin G. Houston and Chris R. Cardwell. Writing, review and editing: Martin G. Houston, Chris R. Cardwell, Úna McMenamin, Brian Johnston, Ronald D. McDowell, Carmel M. Hughes and Peter Murchie. All authors approved the final version of the manuscript.

## CONFLICT OF INTEREST STATEMENT

The authors declare no conflicts of interest.

## Supporting information


**SUPPORTING INFORMATION TABLE S1** Exposure to paracetamol (not combined with weak opioids) and risk of gastrointestinal cancer
**SUPPORTING INFORMATION TABLE S2** Exposure to ibuprofen and risk of gastrointestinal cancer
**STROBE Statement** Checklist of items that should be included in reports of case‐control studies

## Data Availability

The data that support the findings of this study are available from the Institute of Applied Health Sciences, University of Aberdeen. Restrictions apply to the availability of these data, which were used under licence for this study. Data are available from the authors with the permission of the Institute of Applied Health Sciences Section.
